# Shape, Microstructure, and Chemical Composition of Pearls from the Freshwater Clam *Diplodon chilensis* Native to South America

**DOI:** 10.3390/ani13132231

**Published:** 2023-07-07

**Authors:** Gonzalo A. Collado, Moisés A. Valladares, Cristian Suárez, Mathias Seguel, Gerardo Cabello-Guzmán

**Affiliations:** Departamento de Ciencias Básicas, Facultad de Ciencias, Universidad del Bío–Bío, Avenida Andrés Bello 720, Casilla 447, Chillán 3780000, Chile

**Keywords:** bivalves, Chile, mollusks, natural pearls, Raman spectra

## Abstract

**Simple Summary:**

Natural pearls of *Diplodon chilensis*, a freshwater clam native to southern South America, are reported and characterized for the first time. The finding also constitutes the first record of pearls in a species of the genus *Diplodon*. The pearls have different shapes and sizes, and were found in both, male and female specimens. The microstructure and chemical composition of pearls is consistent with those reported in other bivalve species.

**Abstract:**

The capability to produce pearls is widespread in the phylum Mollusca, including bivalves of the superfamily Unionoidea. Here, we identified and characterized natural pearls formed by *Diplodon chilensis*, a freshwater clam native to southern South America, using samples obtained from two lakes located in the Chilean Patagonia. Pearls were studied using light and scanning electron microscopy (SEM), energy-dispersive X-ray spectroscopy (EDX), Fourier transform infrared spectroscopy (FTIR), and Raman spectroscopy. Naturally formed pearls were found in both male and female *D. chilensis* specimens. Pearls are produced in different shapes, including spherical, ellipsoidal, buttoned, and bumpy, ranging in size from 200 µm to 1.9 mm. The internal microstructure is composed of irregular polygonal tablets, about 0.40 to 0.55 μm in thickness. EDX analysis showed that pearls are composed of calcium carbonate. FTIR and Raman spectra recorded several peaks attributable to the aragonite in pearls of this species, as has been shown in other mollusks. In addition to these results, pearls of different colors are illustrated.

## 1. Introduction

Mollusks represent the second group with the largest number of species after the arthropods, reaching a diversity of between 85,000 and 120,000 species [1,2]. In addition to this high diversity, mollusks have a great variety of body plans [3], which has allowed them to colonize different environments across all climatic zones. Since ancient times, these animals have been used as food sources by humans [4,5], as demonstrated by the large number of archaeological records around the world [6,7].

Traditionally, many species of mollusks have been useful for human societies in religious ceremonies, folk medicine, building tools, the production of buttons, fertilizers, cattle feed, decoration, and ornamental purposes [8,9,10,11,12,13]. Moreover, shellfishing and cultured pearl production have been and continue to be important sources of income for the inhabitants of different countries, and in many parts of the world, some species have been included in commercial aquaculture [14,15].

Several species within the classes Bivalvia, Gastropoda, and Cephalopoda can produce pearls, mainly bivalves. The soft bodies of oysters, clams, mussels, and snails are covered by the mantle, a thin epithelial tissue that covers the body organs and secretes the molluscan shell, which is comprised of calcium carbonate and made up of two to five different layers of calcite and/or aragonite [3,16]. In a similar way, molluscan pearls are produced by the mantle when a foreign body is introduced, either accidentally or deliberately, between this tissue and the shell. When this occurs, the outer mantle epithelium envelops the invading object and secretes calcium carbonate to cover it, forming the pearl [3]. In bivalves, pearl microstructure may consist of calcite, aragonite, or vaterite, three polymorphs of calcium carbonate, or a combination of these phases [17,18,19,20,21,22].

Two types of pearls have been described in mollusks: ampullae, also called “half-pearl”, “blister”, or “mabe”, a protuberance of the internal shell surface; and encysted, formed around a foreign object inside the body of the mollusk [23,24,25]. In the latter case, a donor specimen and another recipient are required to produce a pearl. The object to be implanted can be a piece of mantle tissue alone or a small bead made of different solid materials such as shells, corals, and fish scales, among others, together with a piece of mantle tissue. If the implant is successful, a non-nucleated pearl will be produced in the first case and a nucleated pearl in the second [25].

The genus *Diplodon* Spix, 1827, a representative of the family Hyriidae Swainson, 1840, comprises conspicuous freshwater clams that inhabit lakes, rivers, and streams in South America [26]. Although the number of species in the genus is not known with certainty, it contains more than 50 valid species [27,28,29]. To our knowledge, no pearls have been reported in any *Diplodon* species so far. In the study of the shells and pearls carried out in several species of unionids [30], no pearls were found in the subspecies *Diplodon chilensis* (Gray, 1828). In the present investigation, we apply different techniques for the characterization of pearls discovered in populations of this species sampled in Chile. 

A considerable number of studies have been carried out regarding the morphology, taxonomy, ontogeny, karyology, reproduction, life cycle, toxicology, and ecology of *D. chilensis*, which have led to the species being considered the “best known species of Hyriidae in the continent” [31]. The objective of this study is to report the finding of pearls in populations of *D. chilensis* from two Patagonian lakes in southern Chile. The pearls were characterized using a light microscope, a scanning electron microscope, and FTIR and Raman spectroscopy. 

## 2. Materials and Methods

**Figure 1:**
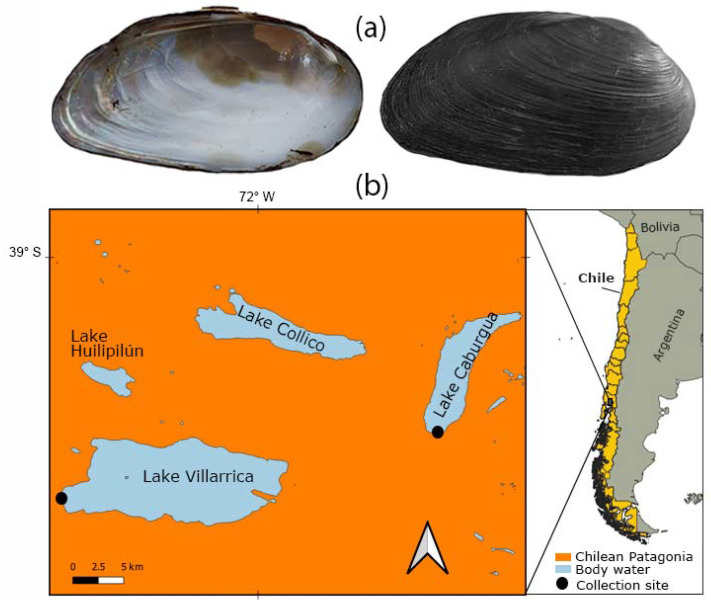
The freshwater clam *Diplodon chilensis* in southern Chile: (**a**) shells (6.7 cm) in internal (**left**) and external (**right**) view; (**b**) collection sites. The maps were created using QGIS Geographic Information System v3.22 (http://www.qgis.org, accessed on 13 September 2022). (Maps: G.A. Collado).

Adult *D. chilensis* individuals were obtained in January 2022 from a shellfisher in Playa Negra, Lake Caburgua (*n* = 100) and by a fisherman at the source of the Toltén River, Lake Villarrica, Southern Chile (*n* = 49) (Figure 1). The clams from both lakes were assigned to *D. chilensis* since (i) our samples were obtained within the species range, (ii) it inhabits Lake Villarrica [32,33], (iii) the current nonexistence of *Diplodon chilensis patagonicus* (d’Orbigny, 1846) populations in Chile [34,35,36,37], and (iv) the absence of characters that differentiate the populations of both lakes. Shell length was measured (in mm) using a vernier caliper (precision 0.01 mm). The clams were dissected using a Motic SMZ–168 stereoscopic microscope and sexed by microscopic examination of gonad smears using a Leica light microscope. The pearls were isolated from the mantle tissue using surgical material, washed with distilled water, and observed using a Hitachi 3500 scanning electron microscope (SEM). SEM was coupled with Bruker model Quantax 100 energy dispersive X-ray spectroscopy (EDX) for the chemical determination of the samples through elemental mapping. Pearls from different individuals were wrapped in paper and then broken with the blunt part of a dissecting needle to observe the internal microstructure using SEM. 

Representative pearls were analyzed with a 4 cm^−1^ resolution using a PerkinElmer Spectrum Two FTIR spectrophotometer. Pearls were also analyzed using the Ocean Onsight Raman spectroscopy model QEPRO-RAMAN-785-PLUS to detect the type of CaCO_3_ polymorph present in the sample. The measurements were acquired with an excitation wavelength of 785 nm at 0.890 nW. The spectra were obtained with an 11 cm^−1^ optical resolution in a spectral range of 0–2000 cm^−1^. 

## 3. Results

**Figure 2:**
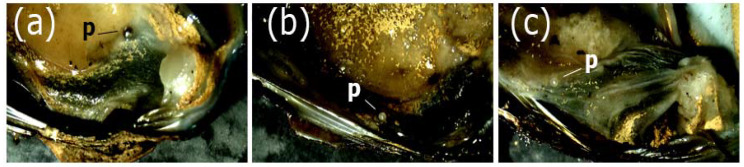
Pearls (p) from the freshwater clam *Diplodon chilensis* from Lake Villarica: (**a**–**c**) pearls of three individuals covered by the mantle. Note a silver pearl in (**a**) and grayish white in (**b**) and (**c**).

**Figure 3:**
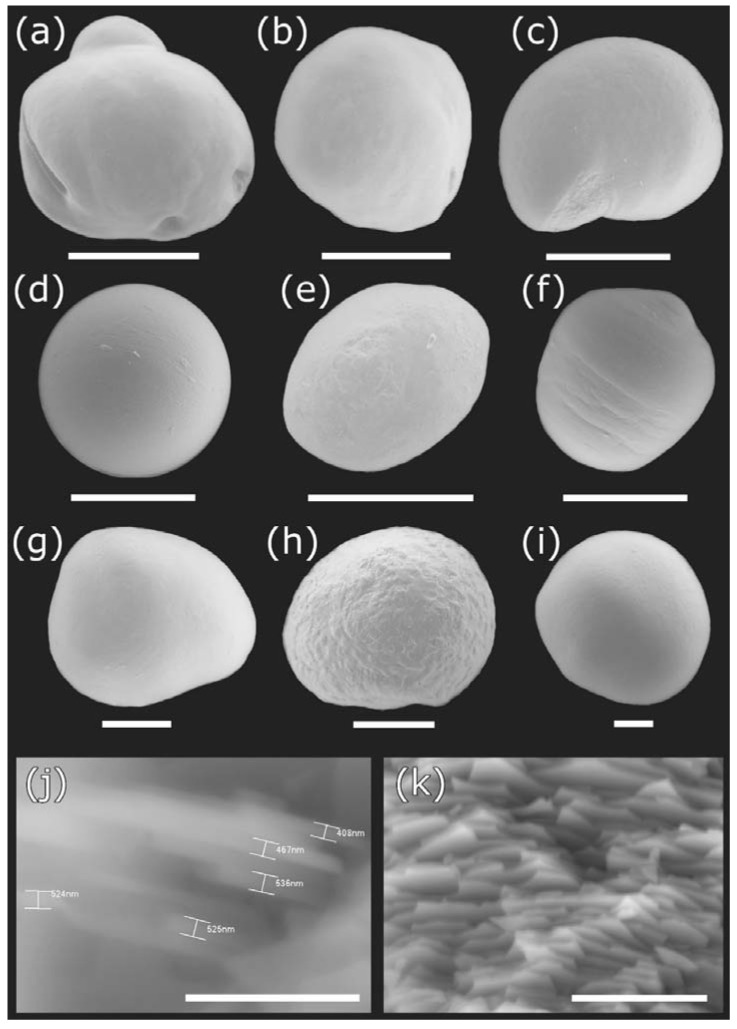
Pearls produced by the freshwater clam *Diplodon chilensis* from southern Chile: (**a**–**h**) pearls obtained from Lake Villarrica; (**i**) pearls obtained from Lake Caburgua; (**j**,**k**) microstructure of pearl tablets seen at different angles and magnifications. Scale Bar: **a** = 1 mm; **b**, **c** = 500 µm; **d**–**f** = 300 µm; **g**–**I** = 200 µm; **j** = 5 µm; **k** = 10 µm.

**Figure 4:**
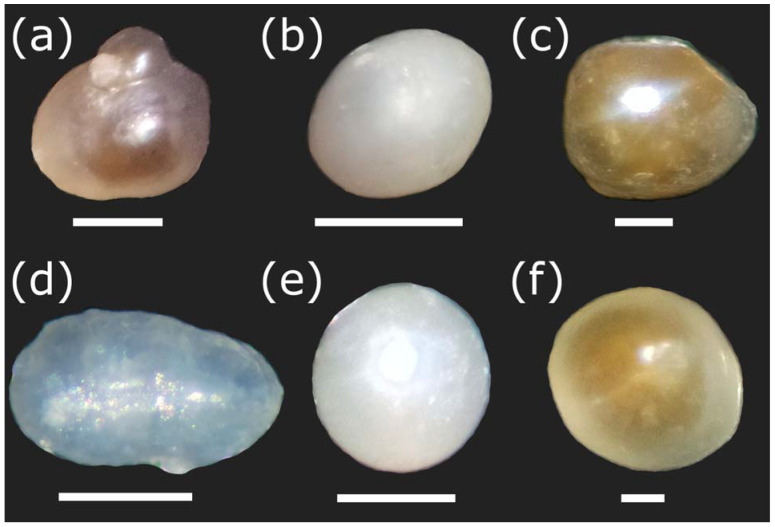
Pearls of *Diplodon chilensis* observed with a stereoscopic microscope showing natural colors. (**a**–**e**) Pearls obtained from Lake Villarrica; (**f**) pearls obtained from Lake Caburgua. Note that the pearls (**a**–**c**,**e**,**f**) were imaged with SEM in Figure 3. In this case, they may have slightly varied in position because different slides were used in both sample viewing techniques. Scale Bar: **a** = 1 mm; **b**, **e** = 300 µm; **c**, **f** = 200 µm; **d** = 500 µm.

Pearls of *D. chilensis* were found covered by the animal’s mantle tissue (Figure 2) and were isolated by making a fine cut on it. Pearls were found in both male and female clams and were of different shapes, including spherical, elliptical, buttoned, or with bumps (“baroque”). With the electron microscope, we observed the external appearance of the pearls, indicating that most of them were smooth (Figure 3). However, some of them had small slits or holes as well as an irregular surface. Most pearls were white or grayish white in color, although a few silvers, light blue, pinkish, or brown were also found (Figure 4). In some clams, there were small pearls in the adductor muscles. The size of the pearls varied between 200 μm and 1.9 mm (Table S1). In Lake Caburgua, we recorded 1% of clams with pearls, while in Lake Villarrica, we recorded 18.4%. In this lake, the smallest individual with a pearl had a length of 41.9 mm. The largest pearl (1.9 mm) was found in a 47.2 mm individual. Only one pearl (924.0 μm) was found in Lake Caburgua in a 65.6 mm individual. 

**Figure 5:**
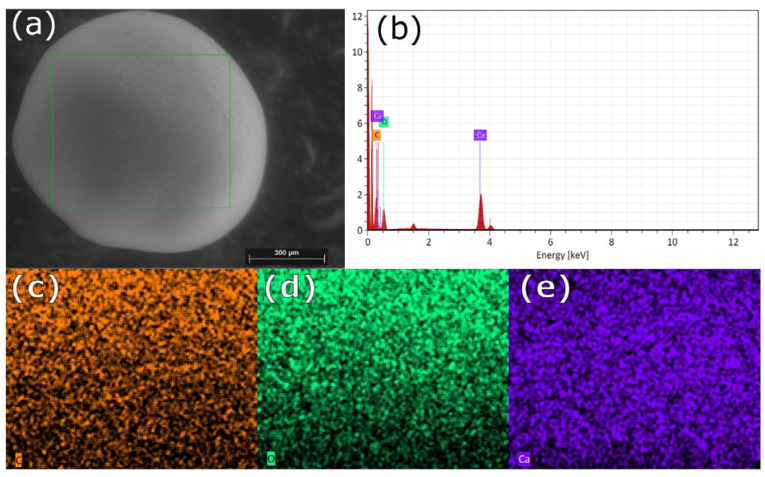
SEM-EDX surface analysis of pearls from *Diplodon chilensis*: (**a**) pearl scanning using SEM; (**b**) EDX spectrum; (**c**) C distribution (orange); (**d**) O distribution (green); (**e**) Ca distribution (violet). In (**a**) the green line frames the analyzed area. The red color in (**b**) shows the energy peaks.

**Figure 6:**
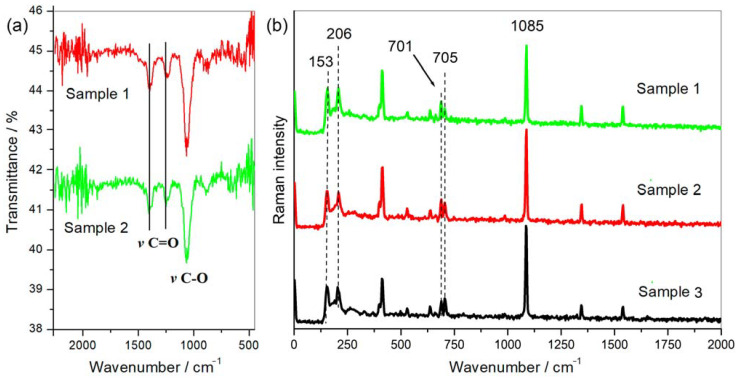
Pearls spectroscopic analysis. (**a**) FTIR and (**b**) Raman spectra obtained from different pearls of *Diplodon chilensis* show the same qualitative and quantitative profile with the characteristic peaks attributed to aragonite [38,39].

Scanning electron microscopy images indicate that the internal microstructure of *D. chilensis* pearls is composed of irregular polygonal tablets about 0.4 to 0.55 μm thick. EDX analysis revealed that they are composed principally of Ca, C, and O in stoichiometric amounts indicative of CaCO_3_ (Figure 5, Table 1 and Tables S2–S4). The FTIR spectrum showed signals located between 1410 and 1255 cm^−1^, at 1065 cm^–1^ and between 2800 and 3000 cm^−1^ (Figure 6). Raman spectra of pearls from different individuals present the main bands at 153, 206, 411, and 1085 cm^−1^ (Figure 6). Other bands of lesser intensity occurred at 701, 705, and 1340–1540 cm^−1^.

## 4. Discussion

In this study, the finding of natural pearls in two lacustric populations of the freshwater clam *D. chilensis* from southern Chile is reported for the first time, also constituting the first report made in the genus *Diplodon*. *Diplodon chilensis* can produce pearls in a variety of shapes, sizes, and colors. Pearl production in this species is not a rare phenomenon since many species of the superfamily Unionoidea Rafinesque, 1820, are potential producers of pearls [40]. However, in South America, only a few species have been described with this capability [23,41,42]. 

In a way, the finding of pearls in *D. chilensis* is quite surprising since this species has been relatively well studied in Chile and Argentina regarding its soft body [43,44,45,46,47,48,49], but until now the finding of gems had not been reported. In our lab, dissections of clams quickly revealed the presence of conspicuous pearls in the mantle tissue of adult individuals of this species. This could be indicative of differences between *D. chilensis* populations in pearl production, and, in fact, our results show that natural pearl formation is more common in Lake Villarrica than Lake Caburgua. It is unknown if these differences between populations in both water bodies have a genetic basis. It has been postulated that variations in certain life history traits between *D. chilensis* populations from lotic and lentic environments could be due to genetic differences [49], although a karyological study contradicted this hypothesis [50]. Consistent with the latter, it has been reported that the populations of this clam from different basins near the Pacific coast in southern Chile are not genetically structured [51], and that differences found in the growth rates can be associated with geographic and limnological parameters [32]. 

Our results suggest that the shell size does not seem to influence the ability to produce pearls because several individuals from Lake Villarrica in the 4.0 to 5.0 cm size class produced pearls, while twice or three times as many individuals from Lake Caburgua between 5.0 and 7.0 cm did not, except for the 65.6 mm specimen. According to Hohn and Costa [42], acidic waters prevent the proper formation of pearls in bivalves, but this would not be the case since the waters of lakes Caburgua and Villarrica have very similar pH [52], although some variation has been reported in this water body [53]. Rahman et al. [54] detected seven species of pearl-producing bivalves in waters off the coast of Bangladesh with a pH of 8.1 to 8.3.

The size of the pearls varies between mollusk species. The largest natural pearl found in the present study measured 1.9 mm. Pearls with similar sizes have been found in other bivalve species from South America with relatively similar shell lengths. *Triplodon corrugatus* (Lamarck, 1819) produces pearls from 2 to 3 mm, *Castalia ambigua* Lamarck, 1819, around 2 mm, and *Prisodon obliquus* Schumager, 1871, from 2.5 to 4 mm [23]. In *Placuna placenta* Linnaeus, 1758, from India, the diameter of pearls varies from 1.5 to 4 mm [54]. The cultured pearls in *Mercenaria mercenaria* (Linnaeus, 1758) and *Pinctada margaritifera* (Linnaeus, 1758) range from 9 to 13 mm, while in *Venerupis* aff. *Decussata* (Linnaeus, 1758), they generally do not exceed 6 mm [55,56]. In the production of cultured pearls, a typical 9-mm round non-beaded pearl takes about 4 years to form [42].

Peaks of the FTIR spectrum located between 1410 and 1255 cm^−1^ have been associated with the vibrations of the carbonyl group (C=O) while that at 1065 cm^−1^ with carbon-oxygen (C-O) confirming the presence of carbonated compounds (CO_3_=) in the samples [57,58]. Signals located between 2800 and 3000 cm^−1^ correspond to carbon–hydrogen (C-H) vibrations that can be attributed to the organic moiety present in the samples [59]. The main peak of pearls in *D. chilensis* obtained by Raman at 1085 cm^–1^ is the main vibration of the CO_3_= molecule in carbonates [60], which is also present in pearls of other mollusk species [22]. Bands at 1084–1087 cm^−1^ have been attributed to aragonite or calcite in different bivalve species [61,62,63,64,65,66]. However, distinguishing between the three carbonate polymorphs (aragonite, vaterite, and calcite) using only the most intense band v1 (symmetric stretching) located approximately at 1085 cm^−1^ is not enough to identify a particular phase [39]. This led some authors to focus on the v2, v3, and v4 vibrational modes of CO_3_= depicting peaks around 850–900, 1430–1600, and 680–750 cm^−1^, respectively, to characterize some of the three phases [39,67]. Thus, the v4 band (a doublet) assigned to the out-of-plane vibrational modes of CO_3_= located at 701 and 705 cm^−1^ and peaks identified in bivalves at 153 and 206 cm^−1^ have been attributed to aragonite [22,39,63,67,68,69,70,71], all of them detected and quantified in *D. chilensis* pearls (Figure 5). No vaterite or calcite could be identified in this clam. 

The causes of the variation in the color of pearls are still a subject of investigation [56,71,72]. Bands obtained near or between 1080, 1135, and 1530 cm^−1^ using Raman analysis, among other bands, suggest that the color of the pearls in several species of bivalves might be due to different chemical compounds, pigments, or optical effects of the sample [56,61,71,72,73,74,75]. However, not all species that have pearls of different colors exhibit these bands [72,76]. On the other hand, there are also other factors involved in the color of pearls, including geographics, genetics, type of mollusk (species), harvest season, water characteristics, depth, quality, and quantity of food (plankton), and the thickness of the pearl [24,42,75,77,78,79,80,81].

To improve the quality of cultured pearls, current trends show that the production of pearls has turned from the frequent improvement of traditional cultivation techniques to producing pearls through selective mollusk breeding [82]. In addition, several studies have detected genes related to the production of pearls in different species of the group, so this economic activity seems to have a very encouraging future [82,83,84]. In this context, the sequencing of the genomes and transcriptomes of some mollusk species [85,86,87,88,89,90] will be important for the identification of genes involved in shell and pearl biomineralization. To date, in *D. chilensis,* no progress has been made in this aspect of its biology.

*Diplodon chilensis*, an efficient filter feeder capable of depleting phytoplankton and bacteria from the water column [91,92,93], is one of the most abundant bivalves distributed in Patagonia [94,95,96]. However, this clam has been considered a threatened species in many Chilean water bodies since various localities in the central-southern parts of the country have experienced a decline in densities and even the disappearance of banks due to anthropic activities [32,50,96,97]. A drastic reduction in population density has also been reported in some lacustric towns in the Argentine Patagonia due to water pollution [98]. Despite these situations, the species has been classified as Least Concern (LC) by the IUNC Red List of Threatened Species as it has a widespread distribution throughout Chile and Argentina [99]. 

The culture of *D. chilensis* has not been implemented in Chile or Argentina. Although the species is not traded for food or any other purpose in the formal markets of Chile, it is edible and sold in informal urban and rural markets in the south of the country, considering the personal observation of the first author and reports by locals [100]. The use of the species as food is also evidenced by the presence of adult shells deposited in pre-Hispanic shell mounds in Patagonia [100,101,102]. The results reported in the present study may be the first step in boosting pearl production on culture farms in the country.

## 5. Conclusions

In this article, we provide a description of the main characteristics of the natural pearls produced by the freshwater clam *Diplodon chilensis*, considering shape, microstructure, and chemical composition. This is the first record of pearls produced in the genus *Diplodon*. Further studies are needed to investigate whether there are population differences in pearl production and culture feasibility for the species.

## Figures and Tables

**Table 1:** Elemental composition of the pearl surface of nine *Diplodon chilensis* individuals obtained by SEM-EDX analysis.

**Element**	**Pearl**								
	1	2	3	4	5	6	7	8	9
Oxygen	38.2	39.4	40.6	39.9	38.4	39.7	40.0	44.5	42.9
Carbon	36.4	36.5	30.8	33.6	32.3	34.8	30.6	35.0	29.8
Calcium	23.1	24.1	27.4	24.8	27.7	24.3	28.9	19.2	27.4

## Data Availability

See Figures S1–S3 and Tables S1–S4. Voucher specimens are housed at the Laboratorio de Malacología y Sistemática Molecular, Universidad del Bío–Bío, Chillán, Chile.

## Supplementary Materials

The following supporting information can be downloaded at: https://www.mdpi.com/article/10.3390/ani13132231/s1. Table S1: Pearls found in *Diplodon chilensis* from southern Chile; Table S2: EDX analysis in a pearl of *Diplodon chilensis* from Lake Villarrica (individual 8, 1 in Table S1); Table S3: EDX analysis in a pearl of *Diplodon chilensis* from Lake Villarrica (individual 12, 2 in Table S1); Table S4: EDX analysis in a pearl of *Diplodon chilensis* from Lake Caburgua (individual 21, 9 in Table S1); Figure S1: SEM–EDX of the pearl’s surface (a) of *Diplodon chilensis* from Lake Villarrica (individual 4); Figure S2: SEM–EDX of the pearl’s surface (a) of *Diplodon chilensis* from Lake Villarrica (individual 12); Figure S3: SEM–EDX of the pearl’s surface (a) of *Diplodon chilensis* from Lake Caburgua (individual 21).
