# Supplementary material for: Shape, Microstructure, and Chemical Composition of Pearls from the Freshwater Clam Diplodon chilensis Native to South America
Source: Animals (Basel). 2023 Jul 7;13(13):2231. doi: 10.3390/ani13132231 (PMC10339977; doi:10.3390/ani13132231)
Supplement: Supplementary file 1 [file animals-13-02231-s001.zip › animals-2222728-supplementary.pdf]

Shape, microstructure and chemical composition of pearls produced by a freshwater clam native to South America

Gonzalo A. Collado*, Moisés A. Valladares, Cristian Suárez, Mathias Seguel and Gerardo Cabello-Guzmán

Departamento de Ciencias Básicas, Facultad de Ciencias, Universidad del Bío-Bío, Avenida Andrés Bello 720, Chillán, Chile

* Corresponding author. E-mail address: gcollado@ubiobio.cl (G.A. Collado).

Table S1. Pearls found in *Diplodon chilensis* from southern Chile.

Lake	Individual	Shell length	Sex	Pearl size (um)
Villarrica	4	52.1	F	200
	12	55.9	M	791
	7	46.1	i	556
	8	47.2	M	1900
	9	51.9	F	900
	14	55.7	M	Not measured
	15	46,1	F	556
	17	48.3	F	Not measured
	34	43,3	M	478; 481
Caburgua	21	65.6	M	924

M: male; F: female; i: indeterminate.

Energy-dispersive X-ray spectroscopy (EDX) analysis coupled to SEM (3 individuals)

Table S2. EDX analysis in a pearl of *Diplodon chilensis* from Lake Villarrica (individual 8, 1 in Table 1).

Element	At. No.	Netto	Mass [%]	Mass Norm. [%]	Atom [%]	abs. error [%] (1 sigma)	rel. error [%] (1 sigma)
Oxygen	8	23090	41.99	44.29	50.47	5.33	12.69
Calcium	20	73734	31.23	32.95	14.99	0.96	3.08
Carbon	6	22816	21.57	22.76	34.54	2.75	12.76
		**Sum**	**94.79**	**100.00**	**100.00**		

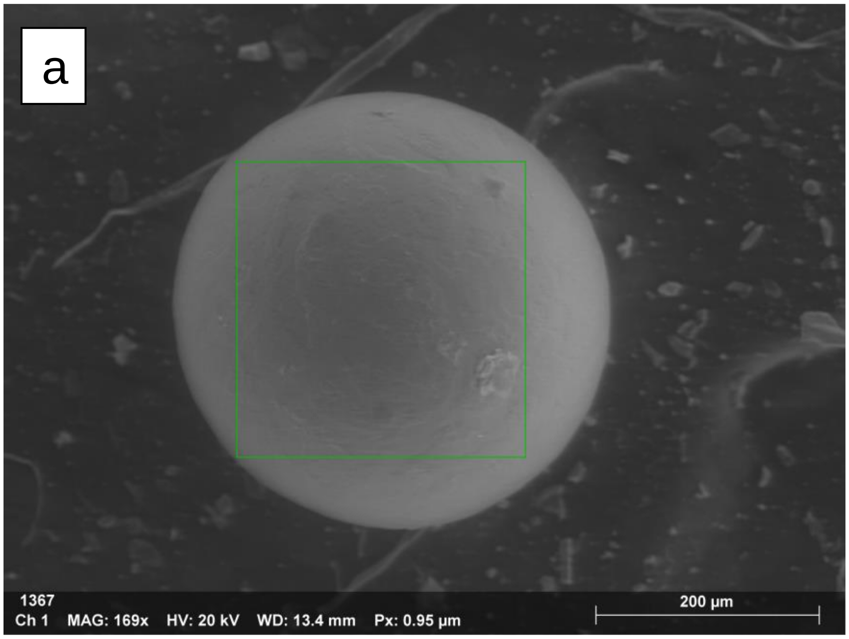

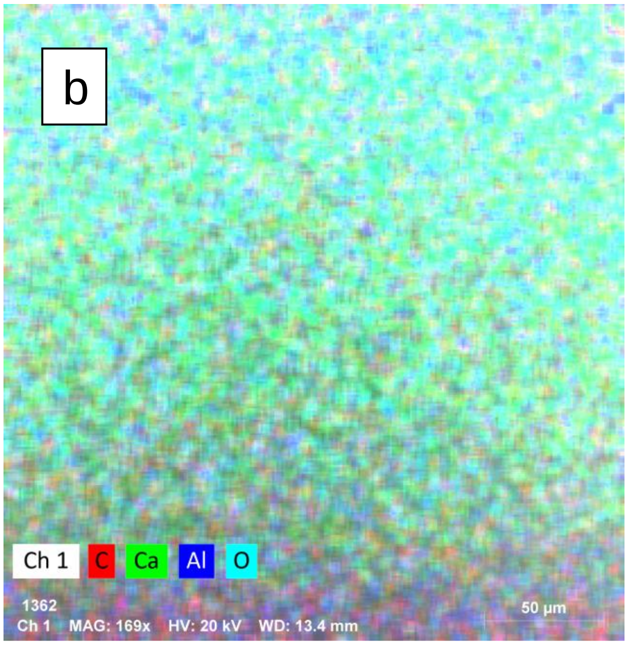
b
Ch 1
C
Ca
Al
O
1362
Ch 1 MAG: 169x HV: 20 kV WD: 13.4 mm
50 µm

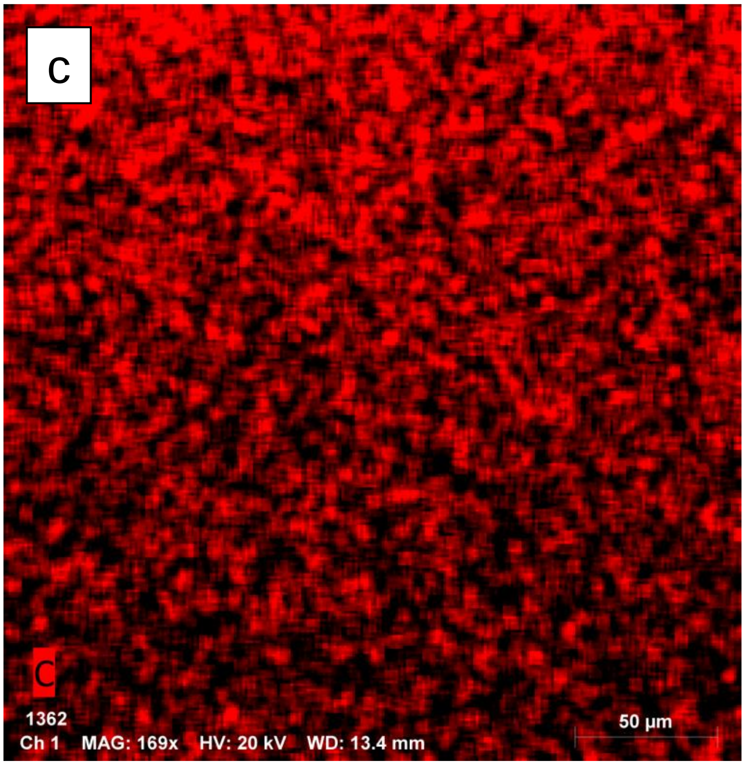
c
C
1362
Ch 1 MAG: 169x HV: 20 kV WD: 13.4 mm
50 µm

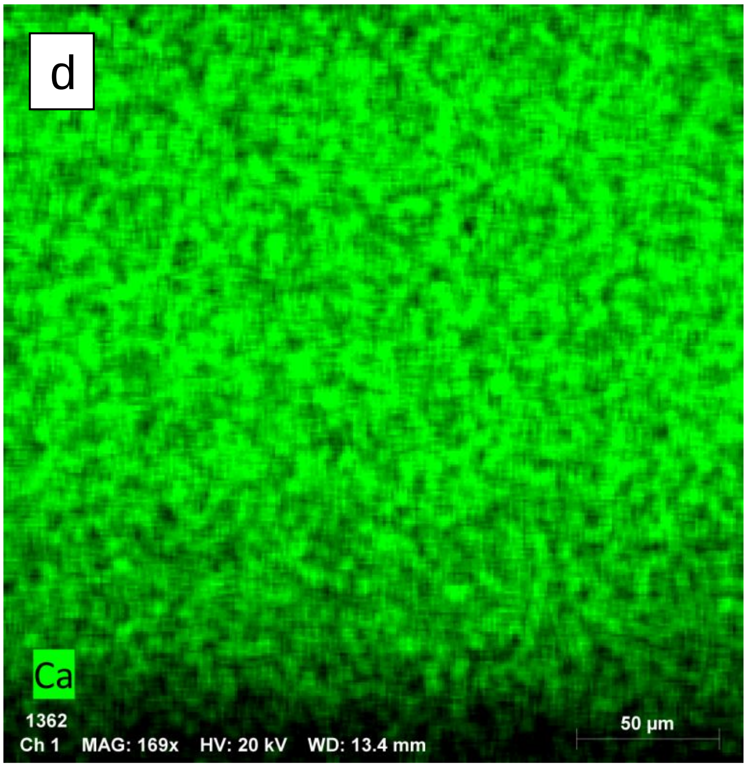
d
Ca
1362
Ch 1 MAG: 169x HV: 20 kV WD: 13.4 mm
50 µm

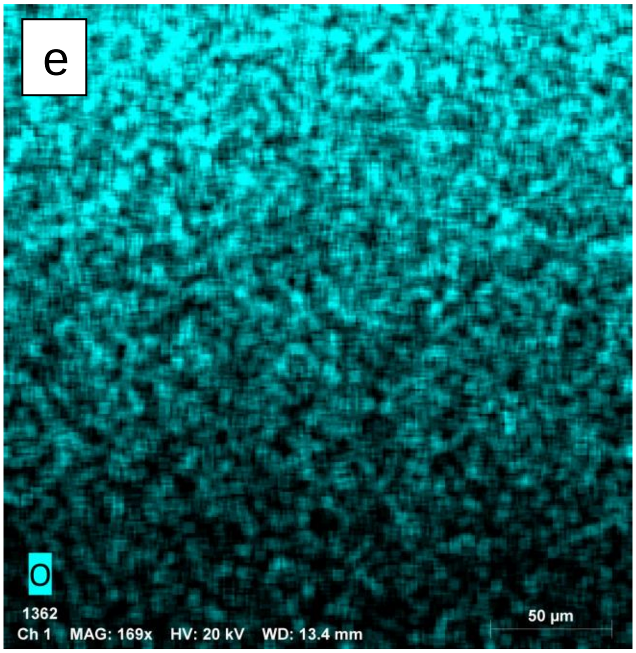
e
O
1362
Ch 1 MAG: 169x HV: 20 kV WD: 13.4 mm
50 µm

f

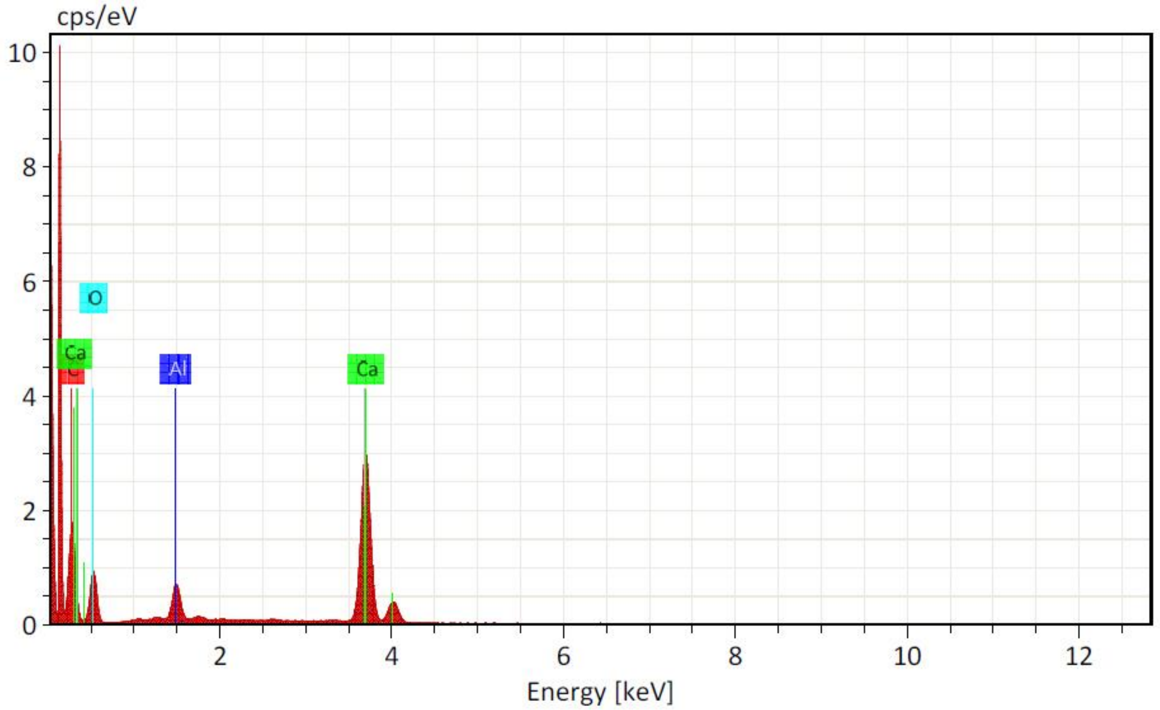

Figure S1. SEM-EDX of the pearl surface (**a**) of *Diplodon chilensis* from Lake Villarrica (individual 4). Presence of elements (C, Ca and O_2) on the pearl surface (**b**). (**c**), while elemental mapping is shown in colors: carbon (red) (**d**), calcium (green) (**e**) and oxygen (blue). Picks of elements are shown in the EDX spectra (**f**).

Table S3. EDX analysis in a pearl of *Diplodon chilensis* from Lake Villarrica (individual 12, 2 in Table 1).

Element	At. No.	Netto	Mass [%]	Mass Norm. [%]	Atom [%]	abs. error [%] (1 sigma)	rel. error [%] (1 sigma)
Oxygen	8	30788	41.88	39.35	40.30	5.17	12.34
Carbon	6	58557	38.86	36.51	49.82	4.55	11.72
Calcium	20	79893	25.69	24.14	9.87	0.80	3.09
		**Sum**	**106.44**	**100.00**	**100.00**		

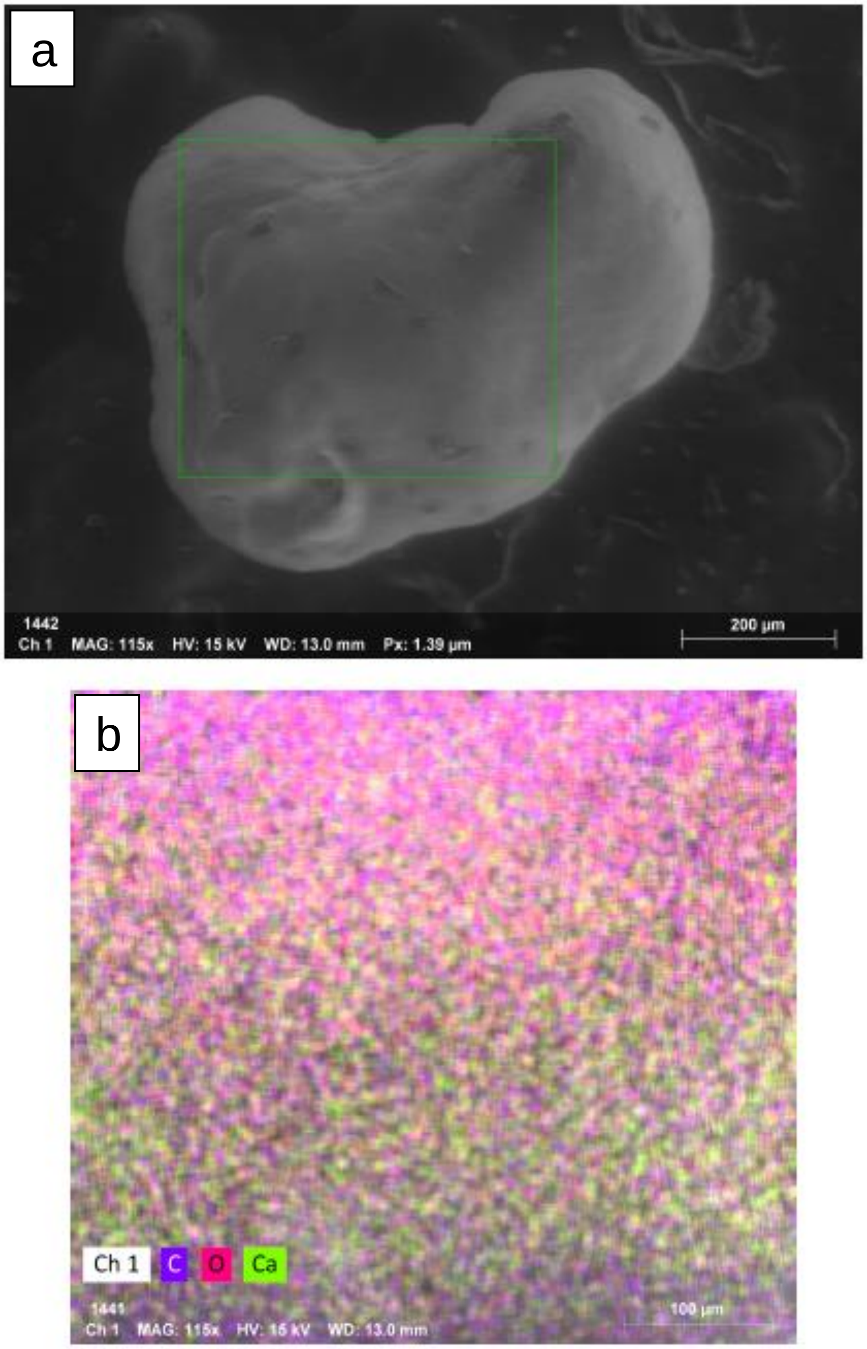
a
1442
Ch 1 MAG: 115x HV: 15 kV WD: 13.0 mm Px: 1.39 µm
200 µm
b
Ch 1
C
O
Ca
1441
Ch 1 MAG: 115x HV: 15 kV WD: 13.0 mm
100 µm

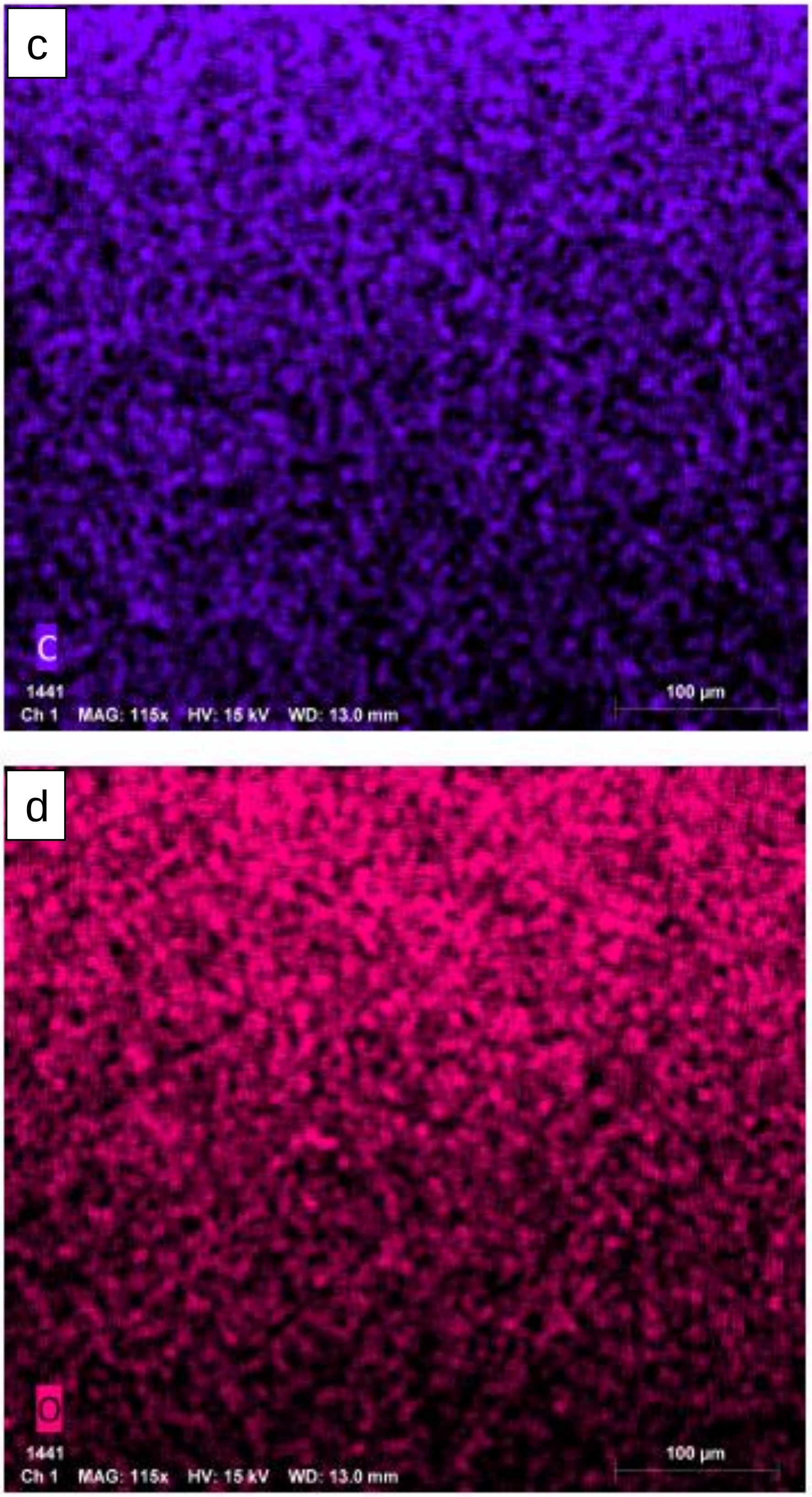
c
C
1441
Ch 1 MAG: 115x HV: 15 kV WD: 13.0 mm
100 µm
d
O
1441
Ch 1 MAG: 115x HV: 15 kV WD: 13.0 mm
100 µm

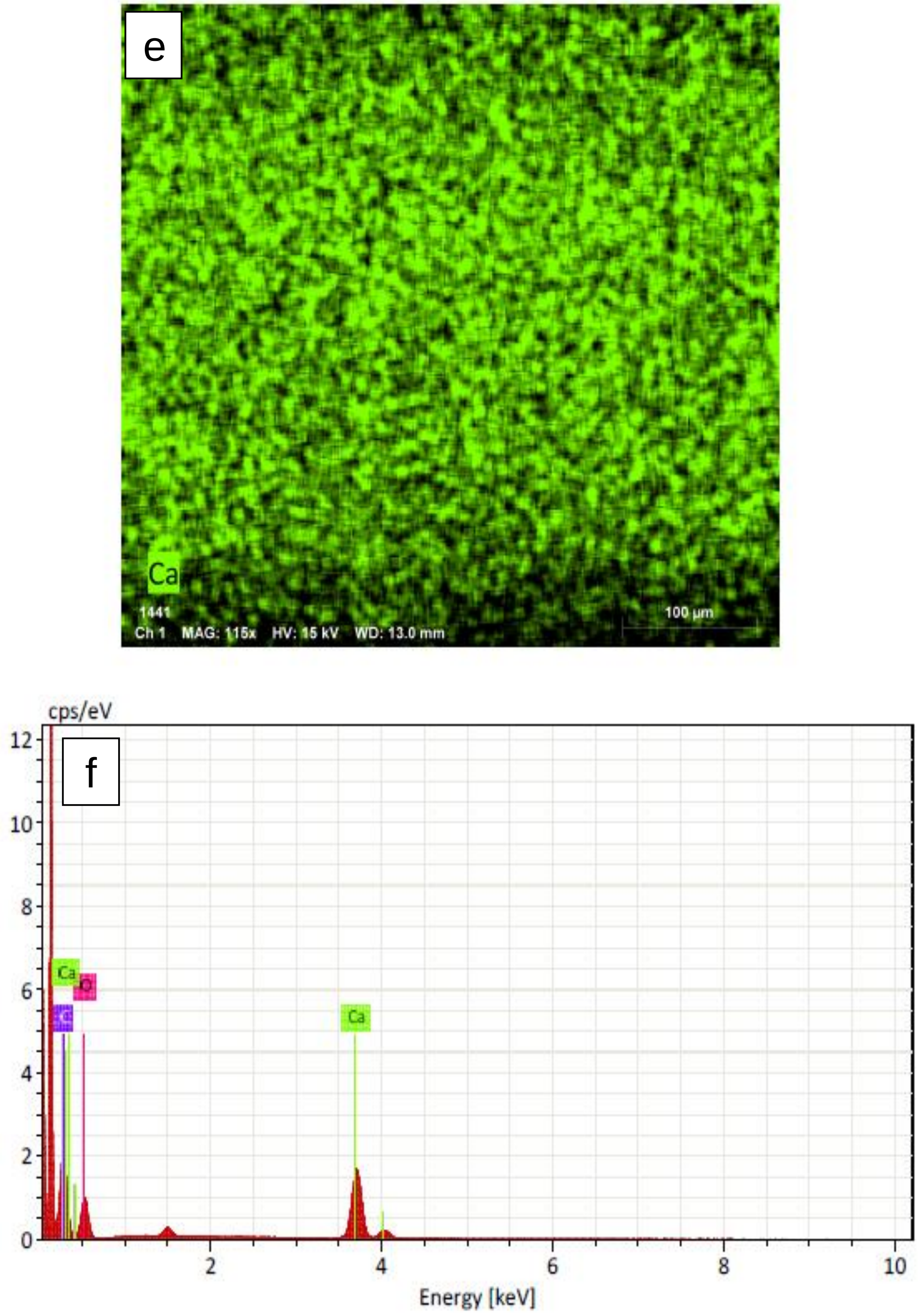

Figure S2. SEM-EDX of the pearl surface (**a**) of *Diplodon chilensis* from Lake Villarrica (individual 12). Presence of elements (C, O_2 and Ca) (**b**) on the pearl surface. Elemental mapping is shown in colors: carbon (violet) (**c**), oxygen (pink) (**d**) and calcium (green) (**e**). Picks of elements are shown in the EDX spectra (**f**).

Table S4. EDX analysis in a pearl of *Diplodon chilensis* from Lake Caburgua (individual 21, 9 in Table 1).

Element	At. No.	Netto	Mass [%]	Mass Norm. [%]	Atom [%]	abs. error [%] (1 sigma)	rel. error [%] (1 sigma)
Oxygen	8	26691	45.42	42.85	45.86	5.68	12.50
Carbon	6	36008	31.56	29.78	42.45	3.85	12.19
Calcium	20	71006	29.00	27.36	11.69	0.90	3.09
		**Sum**	**105.98**	**100.00**	**100.00**		

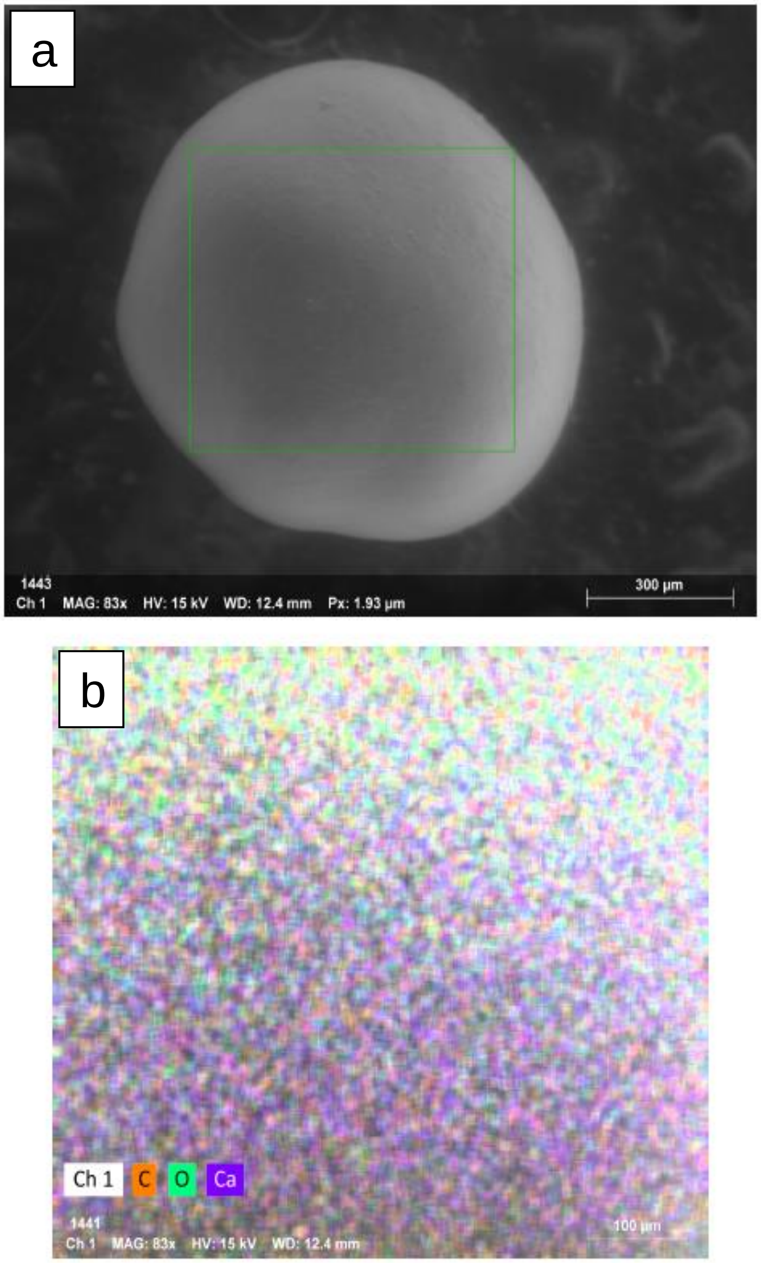

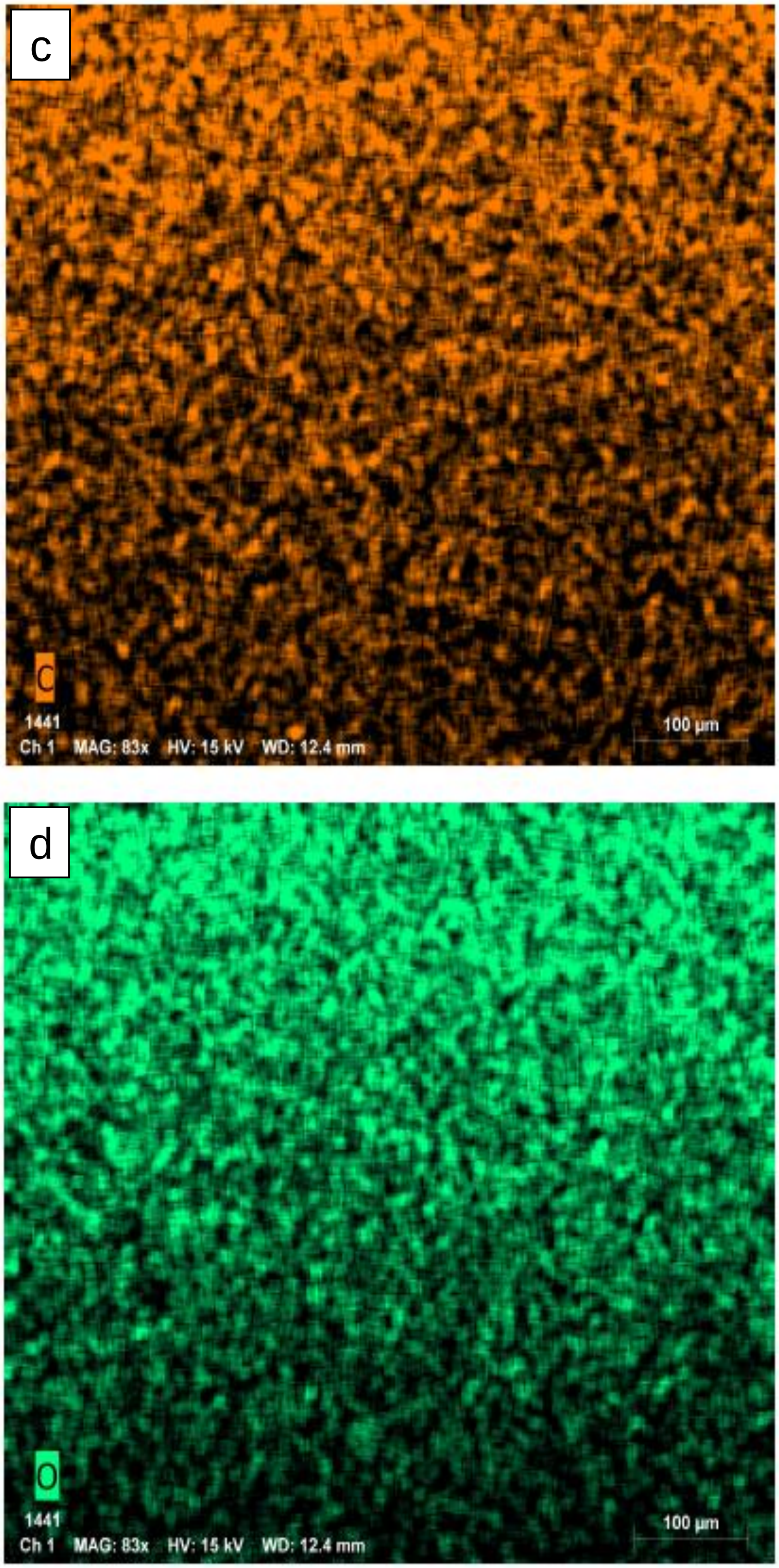
c
C
1441
Ch 1 MAG: 83x HV: 15 kV WD: 12.4 mm
100 µm
d
O
1441
Ch 1 MAG: 83x HV: 15 kV WD: 12.4 mm
100 µm

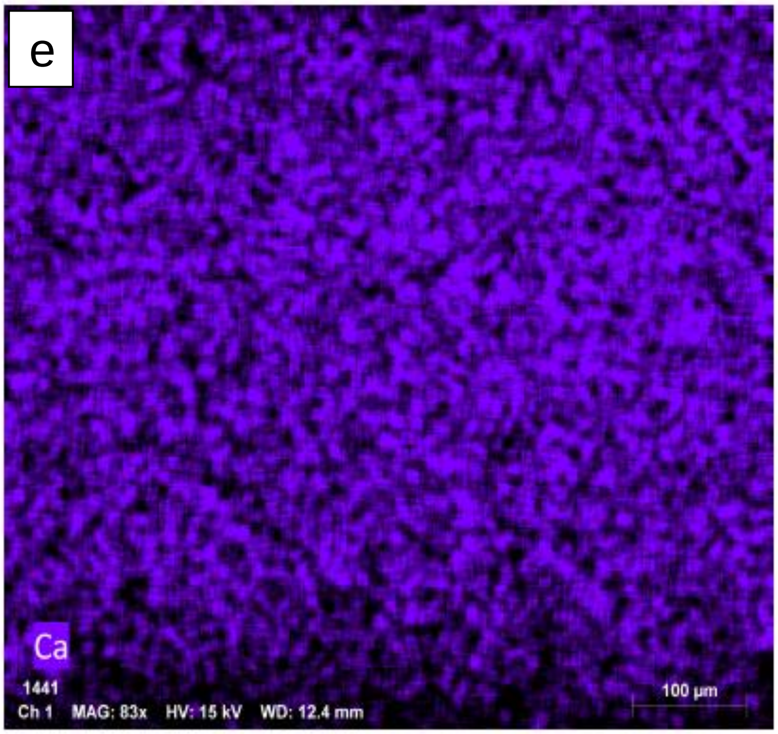

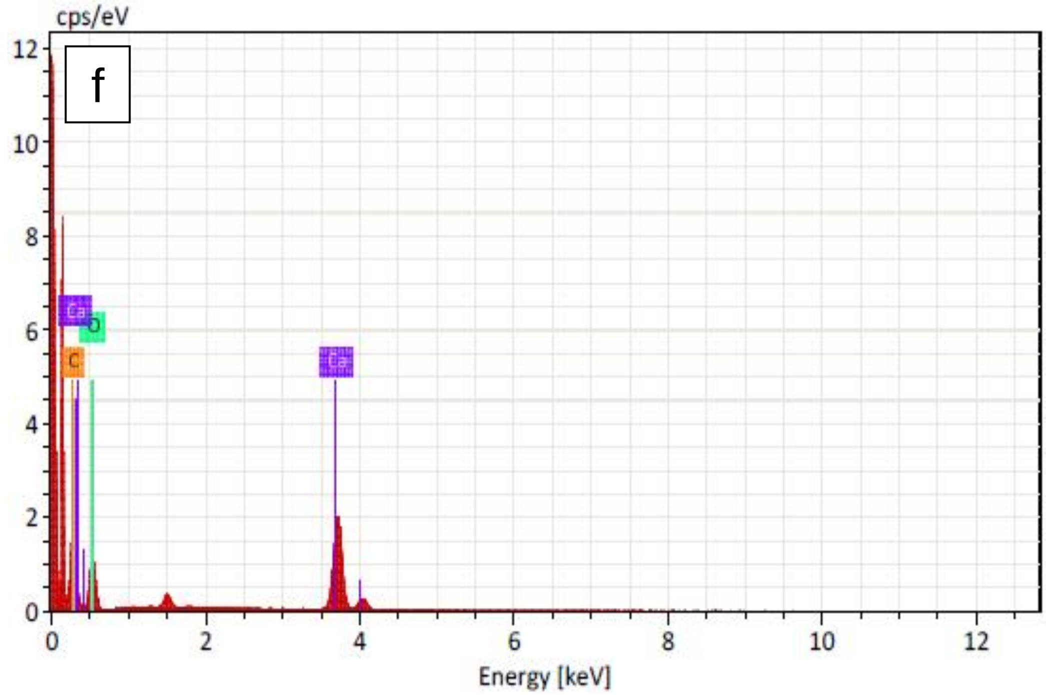

Figure S3. SEM-EDX of the pearl surface (**a**) of *Diplodon chilensis* from Lake Caburgua (individual 21). Presence of elements (C, O_2 and Ca) on the pearl surface (**b**). Elemental mapping is shown in colors: carbon (orange) (**c**), oxygen (green) (**d**) and calcium (violet) (**e**). Picks of elements are shown in the EDS spectra (**f**).
